# Ocular Trauma Trends during COVID-19 Pandemic

**DOI:** 10.21315/mjms2023.30.3.12

**Published:** 2023-06-27

**Authors:** Timothy Khai-Wing Liew, Geng-Yi Yong, Zairah Zainal Abidin, Zalifa Zakiah Asnir

**Affiliations:** Department of Ophthalmology, Ampang Hospital, Selangor, Malaysia

**Keywords:** ocular trauma trends, COVID-19, eye injuries, Malaysia

## Abstract

**Background:**

The article aims to study the demographics and clinical characteristics of ocular trauma patients presenting to the Eye Casualty Clinic between COVID-19 and non-COVID-19 era in Ampang Hospital, Malaysia.

**Methods:**

In this cross-sectional study, data of patients presented with ocular trauma injury to the Ampang Hospital during the COVID-19 era from 18 March 2020 to 17 September 2020 were retrieved and compared with the similar period of the previous non-COVID-19 era year.

**Results:**

Among the total number of 453 patients, 76.82% (*n* = 348) were predominantly males. The commonest age group was between 21 years old–40 years old (49.45%, *n* = 224), and the commonest location of ocular trauma injury occurred at the workplace (38.19%, *n* = 173); welding was the commonest work-related injury (13.83% in 2019; 12.50% in 2020). Injury-to-treatment time was significantly longer during the COVID-19 era, where patients who sought treatment within a day of injury were 27.27% (*n* = 69) in 2019 and 18.50% (*n* = 37) in 2020 (*P* = 0.030). During the COVID-19 pandemic, patients with vision worse than 6/60 on presentation were higher at 8% compared with 3.56% before the COVID-19 pandemic (OR = 2.35; 95% CI: 1.01, 5.48; *P* = 0.047). Similarly, patients with a vision worse than 6/60 post-treatment during the COVID-19 period were significantly higher at 7.00% compared with 1.58% before the COVID-19 pandemic (OR = 4.72; 95% CI: 1.53, 14.62; *P* = 0.007).

**Conclusion:**

The majority of ocular trauma cases in this study population were male adults between 21 years old and 40 years old, and welding was the commonest work-related injury. COVID-19 era has a higher percentage of patients presented with severe visual impairment, longer injury-to-treatment time and poorer post-treatment visual outcomes.

## Introduction

Coronavirus disease 2019 (COVID-19) was declared by the World Health Organization (WHO) as a public health emergency of international concern that was declared as a pandemic on 11 March 2020 (1). It is an infectious disease with high virulence that can lead to respiratory illness with symptoms such as flu, cough, fever and in more severe cases, difficulty in breathing (2). Additionally, in selected groups of people, such as the elderly, those with chronic diseases or immunocompromised people, it can lead to severe acute respiratory infection (SARI), cytokine storm and even multi-organ failure. The threat of COVID-19 was first detected in Malaysia at the end of January 2020. It spread to the entire country and created a worrying situation among the citizens. Thus, in line with an international measure to contain the spread of this disease, Malaysia officially implemented a nationwide Movement Control Order (MCO) on 18 March 2020 as a preventive measure in response to the COVID-19 pandemic (3).

The Ministry of Health (MOH) in Malaysia prioritised essential services such as the emergency department, internal medicine, infectious disease, laboratories and radiology department (4). However, the majority of non-essential healthcare services were disrupted and services were kept at a bare minimum with a limited number of healthcare personnel (5).

In line with this, it may have had a direct impact on the risk and pattern of ocular trauma in the ophthalmology setting. As ocular trauma is a significant global health problem, it is also the leading cause of monocular blindness (6). Reports have shown that about 55 million eye injuries occur each year, of which 1.6 million are blind from injuries (7). But at the same time, ocular trauma is also one of the preventable causes of visual impairment (8).

There were several studies reported in the USA, Italy and India, showing types of ocular trauma injuries and their trend concerning the COVID-19 pandemic period (9–11). However, there is a lack of data found in Southeast Asia.

This study describes the demographics and clinical characteristics of ocular trauma patients presenting to the eye casualty in the COVID-19 era versus the non-COVID-19 era in Ampang Hospital, Selangor, Malaysia.

## Methods

This study was a cross-sectional study conducted in Ampang Hospital, Selangor, Malaysia. It was done with the approval of the Medical Review and Ethics Committee and in concordance with the Declaration of Helsinki. Data was collected using the integrated electronic hospital information system (EHIS) of Ampang Hospital. All patients who presented with ocular trauma injury to the Ampang Hospital from 18 March 2020 to 17 September 2020 (MCO period) were identified and compared with those presented from 18 March 2019 to 17 September 2019 (pre-MCO period). Patients with previous eye injuries or presenting for follow-up reviews were excluded.

Case records from the EHIS data system provided information including demographic, date and duration of injury, ocular trauma diagnosis, mechanism of injury, location of the injury, treatment modalities, and pre- and post-treatment vision. Data were extracted and entered into a data collection form. Patients with incomplete data were contacted via phone to obtain the missing information. The location and mechanism of ocular trauma were jointly classified into the workplace, home, public, road, school and sports centre. The traumas were categorised into open and closed globe injuries according to the Birmingham Eye Trauma Terminology classification (12). All treatments offered to the patients were recorded. Surgical treatment is defined as a procedure that involved cutting off a patient’s tissue or stitching up a previously sustained wound. Some of its examples are corneoscleral laceration repair, globe exploration, lens extraction, anterior chamber washout and intraocular foreign body removal. Non-surgical treatment included eye irrigation, superficial foreign body removal and pharmacological intervention. Pre- and post-treatment best-corrected visual acuity was recorded and categorised according to the WHO classification of visual impairment (13).

All data were analysed using SPSS redundant version 26.0 (Armok, New York, USA) and statistical significance was a two-sided *P*-value of less than 0.05, using chi-squared test or Fisher’s exact test (univariate analysis). Crude odds ratios (ORs) and 95% confidence intervals (CIs) were computed to evaluate the strength of association between various factors (e.g. gender, nationality, ethnicity, age, injury-to-treatment time, location, management, and vision pre- and post-treatment) using cases with no significant difference as the comparison group. For significant factors, the adjusted odds ratio (AOR) was used for multivariate analysis using multinomial logistic regression.

## Results

A total of 453 patients were included in this study. Male patients constituted 76.82% (*n* = 348) of the cases, making the male-to-female ratio 7:2. Malay patients were the most commonly affected population by ocular trauma at 54.30% (*n* = 246). There were 21.41% non-Malaysian who suffered from ocular trauma (Table 1). Among the non-Malaysians, Bangladeshi patients sustained the most ocular trauma at 6.84% (*n* = 31), followed by Burmese patients at 5.52% (*n* = 25).

The age distribution of the patients is shown in Table 1. The mean (SD) age of the patients included in this study was 34.75 (18.63) years in the year 2019 and 33.64 (18.66) in the year 2020. The majority of patients (49.45%, *n* = 224) were 21 years old to 40 years old.

The most common location for ocular trauma injury was at the workplace (38.19%), followed by home-related injuries (30.68%). Welding was the most common work-related injury, which accounted for 13.83% in 2019 and 12.50% in 2020. For home-related injuries, 10.28% of patients in 2019 and 12.00% in 2020 sustained ocular trauma injuries due to mechanical falls. Domestic violence was also recorded with six patients each for both study years (Table 2).

Most of the ocular trauma injuries involved the anterior segment of the eye, 59.83% (*n* = 210) in 2019 and 58.12% (*n* = 161) in 2020. Lids and orbital injuries comprised 34.73% (*n* = 122) in 2019 and 37.18% (*n* = 103) in 2020 whereas posterior segment injuries were the least which accounted 5.41% (*n* = 19) in 2019 and 3.97% (*n* = 11) in 2020 (Table 3). According to the BETTS classification, there were 3.16% (*n* = 8) open globe injuries in 2019 compared to 4.50% (*n* = 9) in 2020 (Table 1).

The majority of the ocular trauma injuries [150 patients (59.29%) in 2019 and 138 patients (69.00%) in 2020] were treated at our centre 1–3 days after the injury. During the pre-COVID-19 period, more patients were treated significantly earlier. Patient who sought treatment within a day of injury were 27.27% (*n* = 69) in 2019 and 18.50% (*n* = 37) in 2020, respectively, (*P* = 0.03) (Table 1).

During the COVID-19 pandemic period, visual acuity on presentation and post-treatment were significantly worse compared with the pre-COVID-19 period. Patients with visual acuity worse than 6/60 on a presentation during the COVID-19 period were 8.00% compared with 3.56% before the COVID-19 pandemic (OR = 2.35; 95% CI: 1.01, 5.48; *P* = 0.047). Similarly, patients with visual acuity worse than 6/60 post-treatment during the COVID-19 period were 7.00% compared with 1.58% before the COVID-19 pandemic (OR = 4.72; 95% CI: 1.53, 14.62; *P* = 0.007) (Table 1).

## Discussion

The nationwide MCO implemented by Malaysia brought many changes to our healthcare system, as well as to citizens seeking medical care. There was a 20.9% reduction in patients presented to the casualty clinic during the COVID-19 pandemic era compared to the pre-pandemic period. This reduction was due to the stay-at-home government order and the fast-spreading ability of the disease, which discourages people from leaving the house, more so coming to the hospital. Similar trends were found in previous studies internationally (9, 10, 11).

We found that majority of patients who presented with ocular trauma injury to our centre were predominantly males. Males were still the breadwinner among most Malaysian families and were involved in the essential sectors allowed to operate during MCO, such as manufacturing, construction and agricultural sectors. The findings were consistent with various studies from Malaysia, India, USA and Italy (2, 9–11). The young working adult age group of 21 years old–40 years old contributed the most cases, with a mean age of 33.63 years old. This result was consistent with a previous study by Yong et al. (14). The majority of patients in our study were Malaysian, where Malay ethnicity comprised the most at 54.30%. Among the non-Malaysian, Bangladeshi and Burmese contributed the highest number of ocular trauma cases. This corresponds with the Labour Force Surveys by the Department of Statistics Malaysia, which states that around 15.00% of the total labour force were foreigners (15). The World Bank 2019 also reported that one-third of workers in the services sector and 25.00% in agriculture are migrants (16).

Connie et al. (10) reported that most ocular traumas happened at home during the COVID-19 pandemic. This was consistent with our study, where we found out that 34.50% of patients sustained home-related injuries during the COVID-19 era as compared to 27.67% during the pre-COVID-19 era. However, our study showed no statistically significant difference in places of ocular trauma injury between COVID-19 and the pre-COVID-19 period. This could be possibly due to the relaxation of MCO rules when Malaysia entered the recovery phase, where nearly all economic sectors were opened, and people could move around more freely.

There was also a reduction in sports-related eye injuries during the COVID-19 era (3.56% in 2019; 1.50% in 2020) as all sports and recreational activities in the area placed under MCO had been suspended (17). Similarly, we also saw a reduction in motor vehicle accident cases during the MCO period (21.34% in 2019; 17.00% in 2020) as traveling on the road was limited to those with valid reasons.

Visual outcomes depend on the types of eye injury, presenting visual acuity and injury-to-treatment time. Our study showed that during the COVID-19 pandemic period in 2020, there were 31 cases (15.50%) that required surgical treatment. Among them, nine cases (4.50%) were open globe injuries. In our study, patients treated within 24 h were significantly lesser in 2020 compared to the previous year. This could be possibly explained by a few factors. During the COVID-19 pandemic, discouragement from leaving home and the fear of meeting a police roadblock en route to the hospital made going to the hospital a tough call. Furthermore, a crowded emergency department, accompanied by the need for a COVID-19 swab test before admission, delayed essential sight-saving procedures, which led to a significantly poorer visual outcome. A detailed pre-operative assessment has been described by Yeoh et al. (18), where there was a multilevel approach prior to an ophthalmic surgery to protect the well-being of patients and healthcare workers. A clinical priority level should be established where clinicians can stratify risk according to the types of cases to initiate appropriate treatment.

## Conclusion

This study concluded that the majority of ocular trauma cases during both COVID-19 and non-COVID-19 eras are adult males between 21 years old and 40 years old, and welding is the commonest work-related injury. COVID-19 era has a higher percentage of patients presented with severe visual impairment on presentation, longer injury-to-treatment time, and poorer post-treatment visual outcomes compared with the pre-COVID-19 era. As ocular trauma is a major cause of vision loss and delay in seeking treatment is associated with significantly poorer presenting vision and visual outcomes after the ophthalmologic intervention, patients should be encouraged to seek treatment promptly, even during the COVID-19 pandemic.

## Figures and Tables

**Table 1:** Characteristics of patient presented with ocular trauma before and during COVID-19 pandemic

Factors	*n* (%)		Crude OR (95% CI)	*P*-value	AOR (95% CI)	*P*-value

	2019, *N* = 253	2020, *N* = 200				
Sex			0.061^*^			
Male	186 (73.52)	162 (81.00)	1.00		1.00	
Female	67 (26.48)	38 (19.00)	1.54 (0.98, 2.41)		1.42 (0.90, 2.26)	0.132
Nationality				0.786^*^		
Malaysian	200 (79.05)	156 (78.00)	1.00			
Non-Malaysian	53 (21.95)	44 (22.00)	0.94 (0.60, 1.48)			
Ethnics						
Malay	139 (54.94)	107 (53.50)	1.00	0.742^*^		
Chinese	50 (19.76)	36 (18.00)	1.64 (0.66, 4.08)	0.286^*^		
Indian	11 (4.35)	13 (6.50)	1.07 (0.65, 1.76)	0.792^*^		
Others	53(20.95)	44 (22.00)	1.15 (0.64, 2.07)	0.634^*^		
Age (years old)						
≤ 20	52 (20.55)	37 (18.50)	1.00	0.892^*^		
21–40	122 (48.22)	102 (51.00)	0.98 (0.53, 1.82)	0.952^*^		
41–60	52 (20.55)	38 (19.0)	0.86 (0.43, 1.72)	0.666^*^		
> 60	27 (10.67)	23 (11.50)	0.84 (0.42, 1.68)	0.613^*^		
Injury to treatment time						
< 1 day	69 (27.27)	37 (18.50)	1.00	0.018^*^	1.00	0.030
1–3 days	150 (59.29)	138 (69.00)	2.22 (1.02, 4.81)	0.045^*^	2.12 (0.97, 4.63)	0.058
4–6 days	18 (7.11)	6 (3.00)	1.72 (1.08, 2.72)	0.022^*^	1.63 (1.02, 2.59)	0.041
≥ 7 days	16 (6.32)	19 (9.50)	0.622 (0.23, 1.70)	0.355^*^	0.59 (0.22, 1.63)	0.311
Location						
Workplace	94 (37.15)	79 (39.50)	1.00	0.346^*^		
Home	70 (27.67)	69 (34.50)	0.56 (0.25, 1.25)	0.157^*^		
Public	20 (7.91)	11 (5.50)	0.64 (0.37, 1.10)	0.106^*^		
Road	54 (21.34)	34 (17.00)	0.68 (0.18, 2.50)	0.558^*^		
Sports centre/Recreation park	9 (3.56)	3 (1.50)	0.34 (0.09, 1.30)	0.115^^^		
School	6 (2.37)	4 (2.00)	0.85 (0.55, 1.33)	0.485^^^		
Birmingham Eye Trauma Terminology (BETTS)				0.457^*^		
Open globe injuries	8 (3.16)	9 (4.50)	1.00			
Closed globe injuries	245 (96.84)	191 (95.50)	1.44 (0.55, 3.81)			
Management				0.259^*^		
Non-surgical	223 (88.14)	169 (84.50)	1.00			
Surgical	30 (11.86)	31 (15.50)	1.364 (0.79, 2.34)			
Vision						
i) On first presentation						
Better than 6/18	196 (77.47)	148 (74.00)	1.00	0.134^*^		
6/18 to 6/60	48 (18.97)	36 (18.00)	0.978 (0.99, 0.61)	0.978^*^		
Worse than 6/60	9 (3.56)	16 (8.00)	2.354 (1.01, 5.48)	0.047^*^		
ii) Post-treatment						
Better than 6/18	220 (86.96)	163 (81.50)	1.00	0.026^*^	1.00	0.854
6/18 to 6/60	29 (11.46)	23 (11.50)	1.070 (0.60, 1.92)	0.819^*^	1.02 (0.56, 1.85)	0.954
Worse than 6/60	4 (1.58)	14 (7.00)	4.72 (1.53, 14.62)	0.007^^^	1.494 (0.35, 6.40)	0.589

Notes: Crude OR were calculated using chi-squared test or Fisher’s exact test; AOR were calculated using Multinomial Logistic Regression

**Table 2:** Causes of ocular trauma based on location

Cause of ocular trauma	*n* (%)		*P*^

	2019, *N* = 253	2020, *N* = 250	
Work related			0.520
Welding	35 (13.83)	25 (12.50)	
Grinding	15 (5.93)	11 (5.50)	
Cutting metal	20 (7.91)	17 (8.50)	
Accidental injuries (e.g. fall, hit by object, chemical)	19 (7.51)	16 (8.00)	
Cut grass	0 (0)	2 (1.00)	
Construction	5 (1.98)	8 (4.00)	
Subtotal	94 (37.15)	79 (39.50)	
Home related			0.867
Mechanical fall	26 (10.28)	24 (12.00)	
Burn/Corrosive agents	7 (2.77)	4 (2.00)	
Gardening/Injured by plants	4 (1.58)	3 (1.50)	
Animal related injuries	2 (0.78)	4 (2.00)	
Accidental injuries (e.g. hit by objects, furniture, playing)	25 (9.88)	28 (14.00)	
Domestic violence	6 (2.37)	6 (3.00)	
Subtotal	70 (27.67)	69 (34.50)	
Public Related			0.229
Assaulted/Conflicts excluding domestic violence	12 (4.74)	7 (3.50)	
Fall	4 (1.58)	4 (2.00)	
Accidental injuries (e.g. sand entered eyes)	4 (1.58)	0 (0.00)	
Subtotal	20 (7.91)	11 (5.50)	
Road related			0.692
Motor vehicle accident	45 (17.79)	28 (14.00)	
Foreign bodies	8 (3.16)	6 (3.00)	
Fall	1 (0.40)	0 (0.00)	
Subtotal	54 (21.34)	34 (17.00)	
Sports			
e.g. Basketball, tennis	9 (3.56)	3 (1.50)	
Subtotal	9 (3.56)	3 (1.50)	
School			0.500
Accidental injuries (e.g. hit by pencil)	4 (1.58)	3 (1.50)	
Fall	2 (0.79)	1 (0.50)	
Subtotal	6 (2.4)	4 (2.00)	

Notes: *P*^value calculated using Fisher’s exact test

**Table 3:** Ocular diagnosis according to anatomical location

Ocular diagnosis	*n* (%)		*P*^

	2019, *N* = 351	2020, *N* = 275	
Orbital/Lid injuries			0.318
Orbital fracture	21 (5.98)	12 (4.33)	
Periorbital haematoma	71 (20.23)	62 (22.38)	
Periorbital skin injury	1 (0.28)	4 (1.44)	
Lid abrasion	2 (0.57)	4 (1.44)	
Lid laceration	27 (7.67)	21 (7.58)	
Subtotal	122 (34.73)	103 (37.18)	
Anterior segment injuries			0.161
Subconjunctival haemorrhage	45 (12.82)	34 (12.27)	
Conjunctival abrasion	2 (0.57)	0 (0.00)	
Conjunctival haematoma	0 (0.00)	1 (0.36)	
Conjunctival laceration	14 (3.99)	8 (2.89)	
Burn injury	12 (3.42)	5 (1.81)	
Corneal abrasion	25 (9.97)	34 (12.27)	
Corneal foreign body	81 (23.08)	60 (21.66)	
Corneal ulcer	0 (0.00)	1 (0.36)	
Corneal laceration	6 (1.71)	6 (2.17)	
Scleral laceration	2 (0.57)	0 (0.00)	
Traumatic uveitis	12 (3.42)	1 (0.36)	
Traumatic mydriasis	3 (0.85)	3 (1.08)	
Traumatic hyphema	6 (1.71)	5 (1.81)	
Traumatic cataract	2 (0.57)	3 (1.08)	
Subtotal	210 (59.83)	161 (58.12)	
Posterior segment injuries			0.080
Vitreous haemorrhage	1 (0.28)	0 (0.00)	
Commotio retinae	12 (3.42)	3 (1.08)	
Retinal haemorrhage	1 (0.28)	0 (0.00)	
Retinal detachment	1 (0.28)	2 (0.72)	
Traumatic optic neuropathy	3 (0.85)	2 (0.72)	
Globe rupture	1 (0.28)	4 (1.44)	
Subtotal	19 (5.41)	11 (3.97)	

Notes:

# one patient can have more than one diagnosis;

*P^*value was calculated using Fisher’s exact test

## References

[1] World Health Organization (WHO) (2020). Coronavirus disease (COVID-19) pandemic.

[2] Agrawal D, Parchand S, Agrawal D, Chatterjee S, Gangwe A, Mishra M (2021). Impact of COVID-19 pandemic and national lockdown on ocular trauma at a tertiary eye care institute. Indian J Ophthalmol.

[3] Ministry of Health Malaysia (2020). Recent situation of COVID-19 pandemic in Malaysia.

[4] World Health Organization (WHO) (2021). COVID-19 continues to disrupt essential health services in 90% of countries.

[5] Hashim JH, Adman MA, Hashim Z, Mohd Radi MF, Kwan SC (2021). COVID-19 epidemic in Malaysia: epidemic progression, challenges, and response. Front Public Health.

[6] Mallika P, Tan A, Asok T, Faisal H, Aziz S, Intan G (2008). Pattern of ocular trauma in Kuching, Malaysia. Malays Fam Physician.

[7] Négrel AD, Thylefors B (1998). The global impact of eye injuries. Ophthalmic Epidemiol.

[8] Parver LM, Dannenberg AL, Blacklow B, Fowler CJ, Brechner RJ, Tielsch JM (1993). Characteristics and causes of penetrating eye injuries reported to the National Eye Trauma System Registry, 1985–91. Public Health Rep.

[9] Marco P, Matilde R, Natalie DG, Enrico L, Giuseppe G, Costantino S (2020). Changing trends of ocular trauma in the time of COVID-19 pandemic. Eye (Lond).

[10] Connie W, Samir NP, Thomas LJ (2020). Ocular trauma during COVID-19 stay-at-home orders: a comparative cohort study. Curr Opin Ophthalmol.

[11] Anthony V, Raja N (2020). Demographics and clinical presentation of patients with ocular disorders during the COVID-19 lockdown in India: a report. Indian J Ophthalmol.

[12] Kuhn F, Morris R, Witherspoon CD, Mester V (2004). The Birmingham eye trauma terminology system (BETT). J Fr Ophtalmol.

[13] (2021). Blindness and vision impairment.

[14] Yong GY, Pan SW, Humayun Akhter F, Law TN, Toh TH (2016). Determinant factors of poor visual outcome after ocular trauma: a retrospective study in central Sarawak, Malaysia. Asia Pac J Ophthalmol (Phila).

[15] World Bank (2020). Who is keeping score? Estimating the number of foreign workers in Malaysia. The Malaysian development experience.

[16] World Bank (2019). The World Bank annual report 2019 ending poverty, investing in opportunity.

[17] Bernama (2021). No exercising or sports in MCO areas now. Free Malaysia Today.

[18] Yeoh SH, Yong GY, Husni MA (2022). New norms in ophthalmic surgery during the COVID-19 pandemic: a narrative review from a Malaysia tertiary eye care center. Eur J Ophthalmol.

